# 5-Bromo-3-cyclo­hexyl­sulfinyl-2,4,6-trimethyl-1-benzo­furan

**DOI:** 10.1107/S1600536814003171

**Published:** 2014-02-15

**Authors:** Hong Dae Choi, Pil Ja Seo, Uk Lee

**Affiliations:** a Department of Chemistry, Dongeui University, San 24 Kaya-dong, Busanjin-gu, Busan 614-714, Republic of Korea; b Department of Chemistry, Pukyong National University, 599-1 Daeyeon 3-dong, Nam-gu, Busan 608-737, Republic of Korea

## Abstract

In the title compound, C_17_H_21_BrO_2_S, the cyclo­hexyl ring adopts a chair conformation and the aryl­sulfinyl unit is positioned equatorially relative to the cyclo­hexyl group. The benzo­furan unit is essentially planar, with an r.m.s. deviation of 0.016 (2) Å. In the crystal, mol­ecules are linked by weak C—H⋯O, C—H⋯π and Br⋯π [3.663 (2) Å] inter­actions, resulting in a three-dimensional network. A Br⋯Br [3.6838 (6) Å] contact is observed. The O atom of the sulfinyl group is disordered over two orientations with an occupancy ratio of 0.863 (5):0.137 (5).

## Related literature   

For background information and the crystal structures of related compounds, see: Choi *et al.* (2011*a* ▶,*b* ▶).
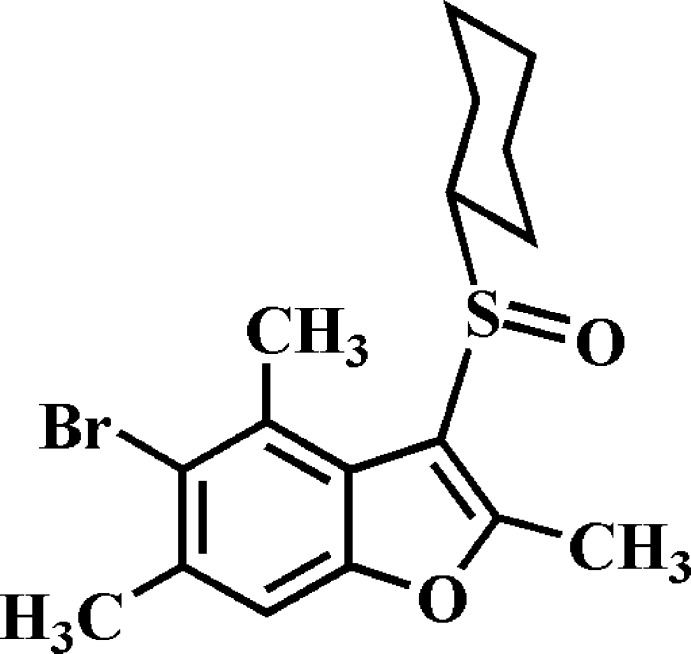


## Experimental   

### 

#### Crystal data  

C_17_H_21_BrO_2_S
*M* *_r_* = 369.31
Triclinic, 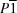

*a* = 5.9051 (4) Å
*b* = 11.7060 (9) Å
*c* = 12.7906 (10) Å
α = 65.839 (4)°
β = 85.795 (4)°
γ = 83.394 (4)°
*V* = 801.01 (10) Å^3^
*Z* = 2
Mo *K*α radiation
μ = 2.70 mm^−1^
*T* = 173 K
0.38 × 0.29 × 0.28 mm

#### Data collection  

Bruker SMART APEXII CCD diffractometer
Absorption correction: multi-scan (*SADABS*; Bruker, 2009 ▶) *T*_min_ = 0.429, *T*_max_ = 0.520
14547 measured reflections
3981 independent reflections
3419 reflections with *I* > 2σ(*I*)
*R*_int_ = 0.038

#### Refinement  

*R*[*F* ^2^ > 2σ(*F* ^2^)] = 0.038
*wR*(*F* ^2^) = 0.091
*S* = 1.05
3981 reflections
203 parameters
4 restraints
H-atom parameters constrained
Δρ_max_ = 1.28 e Å^−3^
Δρ_min_ = −0.88 e Å^−3^

### 

Data collection: *APEX2* (Bruker, 2009 ▶); cell refinement: *SAINT* (Bruker, 2009 ▶); data reduction: *SAINT*; program(s) used to solve structure: *SHELXS97* (Sheldrick, 2008 ▶); program(s) used to refine structure: *SHELXL97* (Sheldrick, 2008 ▶); molecular graphics: *ORTEP-3 for Windows* (Farrugia, 2012 ▶) and *DIAMOND* (Brandenburg, 1998 ▶); software used to prepare material for publication: *SHELXL97*.

## Supplementary Material

Crystal structure: contains datablock(s) I. DOI: 10.1107/S1600536814003171/gg2135sup1.cif

Structure factors: contains datablock(s) I. DOI: 10.1107/S1600536814003171/gg2135Isup2.hkl

Supporting information file. DOI: 10.1107/S1600536814003171/gg2135Isup3.cml

CCDC reference: 986409

Additional supporting information:  crystallographic information; 3D view; checkCIF report

## Figures and Tables

**Table 1:** Hydrogen-bond geometry (Å, °) *Cg*1 is the centroid of the C2–C7 benzene ring.

*D*—H⋯*A*	*D*—H	H⋯*A*	*D*⋯*A*	*D*—H⋯*A*
C12—H12⋯O2*A* ^i^	1.00	2.44	3.355 (3)	151
C11—H11*C*⋯*Cg*1^ii^	0.98	2.83	3.547 (3)	130

Symmetry codes: (i) 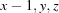; (ii) 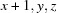.

**Fig. 1.**
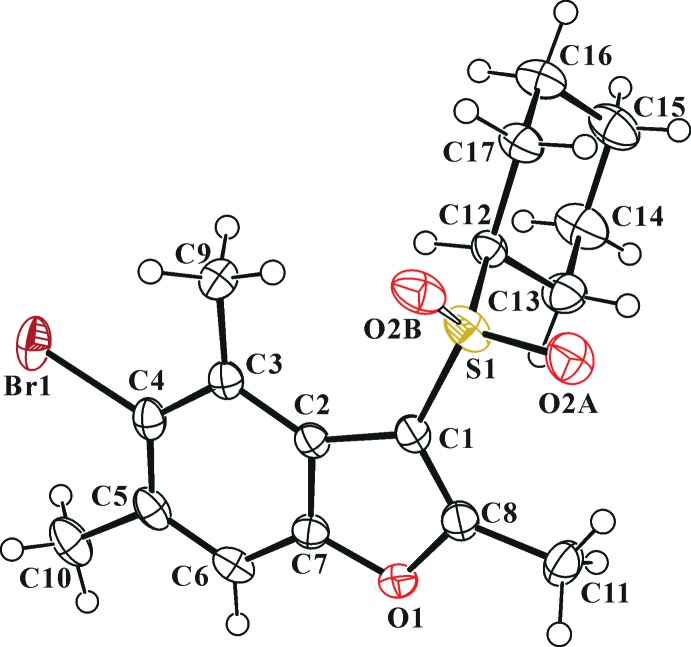
The molecular structure of the title compound with atom numbering scheme. Displacement ellipsoids are drawn at the 50% probability level. H atoms are presented as small spheres of arbitrary radius. The O atom of the sulfinyl group is disordered over two positions with site-occupancy factors, of 0.863 (5) (part A) and 0.137 (5) (part B).

**Fig. 2.**
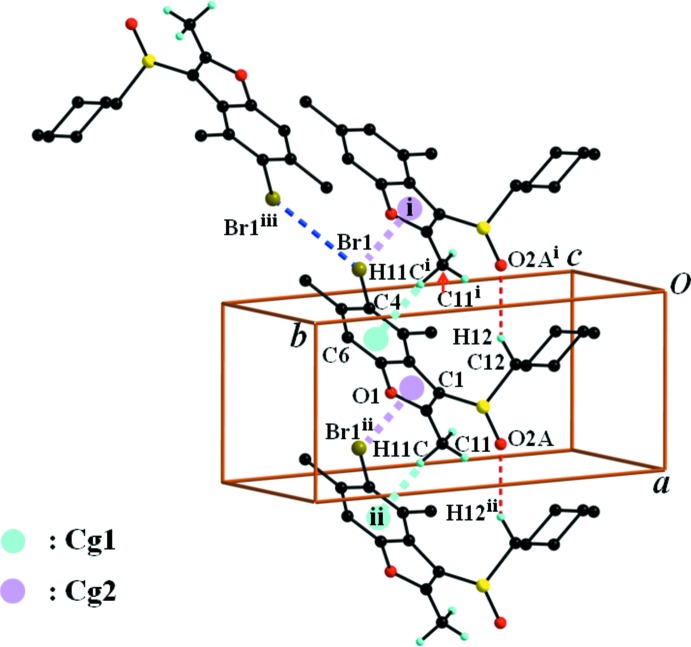
A view of the C—H···O, C—H···π and C—Br···π and Br···Br interactions (dotted lines) in the crystal structure of the title compound. H atoms non-participating in hydrogen-bonding were omitted for clarity. [Symmetry codes: (i) x - 1, y, z; (ii) x + 1, y, z; (iii) - x - 1, - y + 2, - z.]

## supplementary crystallographic information

### 1. Comment

As a part of our ongoing study of 5-bromo-3-cyclohexylsulfinyl-1-benzofuran derivatives containing a methyl group in 2-position (Choi *et al.* , 2011*a*) and methyl substituents in the 2,7-positions (Choi *et al.* , 2011*b*), we report herein the crystal structure of the title compound.

In the title molecule (Fig. 1), the benzofuran unit is essentially planar, with a mean deviation of 0.016 (2) Å from the least-squares plane defined by the nine constituent atoms. The cyclohexyl ring is in the chair form and the arylsulfinyl moiety is positioned equatorial relative to the cyclohexyl group. The O atom of the sulfinyl group is disordered over two positions with site-occupancy factors, from refinement, of 0.863 (5) (part A) and 0.137 (5) (part B). In the crystal structure (Fig. 2), molecules are connected by weak C—H···O and C—H···π hydrogen bonds (Table 1; Cg1 is the centroid of the C2–C7 benzene ring), and by C4—Br1···π interactions between the bromine atom and the furan ring of a neighbouring molecule with a Br1···Cg2^i^ = 3.663 (2) Å (Cg2 is the centroid of the C1/C2/C7/O1/C8 furan ring). The crystal packing (Fig. 2) also exhibits a Br1···Br1^iii^ contact at 3.6838 (6) Å.

### 2. Experimental

3-Chloroperoxybenzoic acid (77%, 224 mg, 1.0 mmol) was added in small portions to a stirred solution of 5-bromo-3-cyclohexylsulfanyl-2,4,6-trimethyl-1-benzofuran (282 mg, 0.9 mmol) in dichloromethane (30 mL) at 273 K. After being stirred at room temperature for 6h, the mixture was washed with saturated sodium bicarbonate solution and the organic layer was separated, dried over magnesium sulfate, filtered and concentrated at reduced pressure. The residue was purified by column chromatography (hexane–ethyl acetate, 2:1 v/v) to afford the title compound as a colorless solid [yield 71%, m.p. 450–451 K; *R*_f_ = 0.46 (hexane–ethyl acetate, 2:1 v/v)]. Single crystals suitable for X-ray diffraction were prepared by slow evaporation of a solution of the title compound in acetone at room temperature.

### 3. Refinement

All H atoms were positioned geometrically and refined using a riding model, with C—H = 0.95 Å for aryl, 1.0 Å for methine, 0.99 Å for methylene and 0.98 Å for methyl H atoms, respectively. *U*_iso_ = 1.2*U*_eq_ (C) for aryl, methine and methylene, and 1.5*U*_eq_ for methyl H atoms. The positions of methyl hydrogens were optimized rotationally. The O atom of sulfinyl group is disordered over two positions with site-ccupancy factors, from refinement of 0.863 (5) (part A) and 0.137 (5) (part B). The distance of S—O sets was restrained to 0.001 Å using command SADI and DELU.

### Crystal data

**Table:**

C_17_H_21_BrO_2_S	*Z* = 2
*M**_r_* = 369.31	*F*(000) = 380
Triclinic, *P*1	*D*_x_ = 1.531 Mg m^−^^3^
Hall symbol: -P 1	Melting point = 450–451 K
*a* = 5.9051 (4) Å	Mo *K*α radiation, λ = 0.71073 Å
*b* = 11.7060 (9) Å	Cell parameters from 6652 reflections
*c* = 12.7906 (10) Å	θ = 3.1–28.4°
α = 65.839 (4)°	µ = 2.70 mm^−^^1^
β = 85.795 (4)°	*T* = 173 K
γ = 83.394 (4)°	Block, colourless
*V* = 801.01 (10) Å^3^	0.38 × 0.29 × 0.28 mm

### Data collection

**Table:**

Bruker SMART APEXII CCD diffractometer	3981 independent reflections
Radiation source: rotating anode	3419 reflections with *I* > 2σ(*I*)
Graphite multilayer monochromator	*R*_int_ = 0.038
Detector resolution: 10.0 pixels mm^-1^	θ_max_ = 28.5°, θ_min_ = 3.1°
φ and ω scans	*h* = −7→7
Absorption correction: multi-scan (*SADABS*; Bruker, 2009)	*k* = −15→15
*T*_min_ = 0.429, *T*_max_ = 0.520	*l* = −17→17
14547 measured reflections	

### Refinement

**Table:**

Refinement on *F*^2^	Primary atom site location: structure-invariant direct methods
Least-squares matrix: full	Secondary atom site location: difference Fourier map
*R*[*F*^2^ > 2σ(*F*^2^)] = 0.038	Hydrogen site location: difference Fourier map
*wR*(*F*^2^) = 0.091	H-atom parameters constrained
*S* = 1.05	*w* = 1/[σ^2^(*F*_o_^2^) + (0.0292*P*)^2^ + 1.0109*P*] where *P* = (*F*_o_^2^ + 2*F*_c_^2^)/3
3981 reflections	(Δ/σ)_max_ = 0.001
203 parameters	Δρ_max_ = 1.28 e Å^−^^3^
4 restraints	Δρ_min_ = −0.88 e Å^−^^3^

### Special details

**Table:**

Geometry. All esds (except the esd in the dihedral angle between two l.s. planes) are estimated using the full covariance matrix. The cell esds are taken into account individually in the estimation of esds in distances, angles and torsion angles; correlations between esds in cell parameters are only used when they are defined by crystal symmetry. An approximate (isotropic) treatment of cell esds is used for estimating esds involving l.s. planes.
Refinement. Refinement of F^2^ against ALL reflections. The weighted R-factor wR and goodness of fit S are based on F^2^, conventional R-factors R are based on F, with F set to zero for negative F^2^. The threshold expression of F^2^ > 2sigma(F^2^) is used only for calculating R-factors(gt) etc. and is not relevant to the choice of reflections for refinement. R-factors based on F^2^ are statistically about twice as large as those based on F, and R- factors based on ALL data will be even larger.

### Fractional atomic coordinates and isotropic or equivalent isotropic displacement parameters (Å^2^)

**Table:**

	*x*	*y*	*z*	*U*_iso_*/*U*_eq_	Occ. (<1)
Br1	−0.27560 (5)	0.86915 (3)	0.02612 (2)	0.03843 (10)	
S1	0.58237 (11)	0.47281 (6)	0.18104 (6)	0.03238 (15)	
O1	0.4834 (3)	0.69082 (15)	0.35369 (15)	0.0281 (4)	
O2A	0.8063 (4)	0.4118 (2)	0.2215 (2)	0.0408 (6)	0.863 (5)
O2B	0.637 (2)	0.5186 (11)	0.0592 (3)	0.036 (3)	0.137 (5)
C1	0.4916 (4)	0.5828 (2)	0.2422 (2)	0.0231 (5)	
C2	0.2955 (4)	0.6766 (2)	0.2109 (2)	0.0222 (4)	
C3	0.1240 (4)	0.7157 (2)	0.1289 (2)	0.0232 (5)	
C4	−0.0359 (4)	0.8119 (2)	0.1324 (2)	0.0264 (5)	
C5	−0.0339 (4)	0.8713 (2)	0.2086 (2)	0.0290 (5)	
C6	0.1401 (4)	0.8330 (2)	0.2854 (2)	0.0290 (5)	
H6	0.1498	0.8709	0.3377	0.035*	
C7	0.2994 (4)	0.7378 (2)	0.2837 (2)	0.0246 (5)	
C8	0.5969 (4)	0.5962 (2)	0.3264 (2)	0.0273 (5)	
C9	0.1167 (4)	0.6589 (2)	0.0433 (2)	0.0292 (5)	
H9A	−0.0024	0.6006	0.0669	0.044*	
H9B	0.2647	0.6131	0.0395	0.044*	
H9C	0.0829	0.7257	−0.0323	0.044*	
C10	−0.2122 (5)	0.9752 (2)	0.2065 (3)	0.0404 (7)	
H10A	−0.1816	1.0035	0.2660	0.061*	
H10B	−0.3633	0.9438	0.2210	0.061*	
H10C	−0.2078	1.0457	0.1313	0.061*	
C11	0.8011 (5)	0.5326 (3)	0.3952 (2)	0.0345 (6)	
H11A	0.8868	0.4773	0.3628	0.052*	
H11B	0.7535	0.4826	0.4745	0.052*	
H11C	0.8980	0.5958	0.3938	0.052*	
C12	0.3727 (4)	0.3601 (2)	0.2543 (2)	0.0236 (5)	
H12	0.2168	0.4061	0.2405	0.028*	
C13	0.4062 (5)	0.2963 (3)	0.3826 (2)	0.0361 (6)	
H13A	0.3831	0.3599	0.4157	0.043*	
H13B	0.5643	0.2562	0.3976	0.043*	
C14	0.2387 (6)	0.1967 (3)	0.4400 (2)	0.0447 (7)	
H14A	0.2694	0.1530	0.5228	0.054*	
H14B	0.0811	0.2381	0.4320	0.054*	
C15	0.2590 (6)	0.1012 (3)	0.3867 (3)	0.0442 (7)	
H15A	0.1432	0.0407	0.4226	0.053*	
H15B	0.4116	0.0538	0.4016	0.053*	
C16	0.2251 (5)	0.1658 (3)	0.2582 (2)	0.0380 (6)	
H16A	0.0671	0.2061	0.2437	0.046*	
H16B	0.2464	0.1021	0.2251	0.046*	
C17	0.3923 (5)	0.2649 (2)	0.1994 (2)	0.0312 (5)	
H17A	0.3592	0.3091	0.1169	0.037*	
H17B	0.5499	0.2237	0.2064	0.037*	

### Atomic displacement parameters (Å^2^)

**Table:**

	*U*^11^	*U*^22^	*U*^33^	*U*^12^	*U*^13^	*U*^23^
Br1	0.02588 (14)	0.03838 (16)	0.03727 (16)	0.00417 (10)	−0.00621 (10)	−0.00237 (11)
S1	0.0252 (3)	0.0267 (3)	0.0457 (4)	−0.0040 (2)	0.0114 (3)	−0.0168 (3)
O1	0.0262 (9)	0.0269 (8)	0.0338 (9)	−0.0042 (7)	−0.0043 (7)	−0.0140 (7)
O2A	0.0242 (10)	0.0408 (13)	0.0592 (16)	0.0009 (8)	0.0026 (9)	−0.0238 (11)
O2B	0.036 (8)	0.037 (7)	0.0463 (14)	−0.002 (5)	0.009 (2)	−0.029 (5)
C1	0.0189 (11)	0.0204 (10)	0.0280 (11)	−0.0024 (8)	0.0018 (9)	−0.0082 (9)
C2	0.0185 (11)	0.0198 (10)	0.0274 (11)	−0.0033 (8)	0.0016 (9)	−0.0086 (9)
C3	0.0201 (11)	0.0214 (10)	0.0247 (11)	−0.0036 (8)	0.0019 (9)	−0.0059 (9)
C4	0.0201 (11)	0.0221 (10)	0.0294 (12)	−0.0022 (8)	0.0003 (9)	−0.0028 (9)
C5	0.0250 (12)	0.0194 (10)	0.0373 (13)	−0.0025 (9)	0.0080 (10)	−0.0075 (10)
C6	0.0321 (13)	0.0223 (11)	0.0348 (13)	−0.0063 (9)	0.0051 (10)	−0.0138 (10)
C7	0.0237 (11)	0.0228 (10)	0.0269 (11)	−0.0059 (9)	0.0002 (9)	−0.0086 (9)
C8	0.0216 (11)	0.0238 (11)	0.0332 (13)	−0.0042 (9)	−0.0006 (9)	−0.0078 (10)
C9	0.0275 (12)	0.0318 (12)	0.0278 (12)	−0.0014 (10)	−0.0037 (10)	−0.0116 (10)
C10	0.0358 (15)	0.0259 (12)	0.0544 (18)	0.0032 (11)	0.0080 (13)	−0.0143 (12)
C11	0.0257 (13)	0.0370 (14)	0.0360 (14)	−0.0034 (10)	−0.0073 (11)	−0.0089 (11)
C12	0.0211 (11)	0.0225 (10)	0.0277 (11)	−0.0013 (8)	0.0014 (9)	−0.0110 (9)
C13	0.0481 (17)	0.0338 (13)	0.0275 (13)	−0.0066 (12)	−0.0022 (11)	−0.0124 (11)
C14	0.068 (2)	0.0347 (14)	0.0303 (14)	−0.0148 (14)	0.0144 (14)	−0.0118 (12)
C15	0.062 (2)	0.0276 (13)	0.0407 (16)	−0.0113 (13)	0.0105 (14)	−0.0115 (12)
C16	0.0459 (17)	0.0315 (13)	0.0416 (15)	−0.0113 (12)	0.0049 (13)	−0.0190 (12)
C17	0.0396 (15)	0.0261 (11)	0.0302 (13)	−0.0045 (10)	0.0038 (11)	−0.0141 (10)

### Geometric parameters (Å, º)

**Table:**

Br1—C4	1.903 (2)	C10—H10A	0.9800
Br1—Br1^i^	3.6838 (6)	C10—H10B	0.9800
S1—O2B	1.450 (2)	C10—H10C	0.9800
S1—O2A	1.451 (2)	C11—H11A	0.9800
S1—C1	1.778 (2)	C11—H11B	0.9800
S1—C12	1.830 (2)	C11—H11C	0.9800
O1—C7	1.373 (3)	C12—C13	1.516 (3)
O1—C8	1.381 (3)	C12—C17	1.531 (3)
C1—C8	1.353 (3)	C12—H12	1.0000
C1—C2	1.457 (3)	C13—C14	1.528 (4)
C2—C7	1.392 (3)	C13—H13A	0.9900
C2—C3	1.411 (3)	C13—H13B	0.9900
C3—C4	1.397 (3)	C14—C15	1.519 (4)
C3—C9	1.501 (3)	C14—H14A	0.9900
C4—C5	1.412 (4)	C14—H14B	0.9900
C5—C6	1.379 (4)	C15—C16	1.520 (4)
C5—C10	1.506 (3)	C15—H15A	0.9900
C6—C7	1.379 (3)	C15—H15B	0.9900
C6—H6	0.9500	C16—C17	1.526 (4)
C8—C11	1.482 (3)	C16—H16A	0.9900
C9—H9A	0.9800	C16—H16B	0.9900
C9—H9B	0.9800	C17—H17A	0.9900
C9—H9C	0.9800	C17—H17B	0.9900
			
C4—Br1—Br1^i^	129.48 (8)	H10B—C10—H10C	109.5
O2B—S1—O2A	97.6 (6)	C8—C11—H11A	109.5
O2B—S1—C1	119.2 (5)	C8—C11—H11B	109.5
O2A—S1—C1	110.03 (13)	H11A—C11—H11B	109.5
O2B—S1—C12	123.9 (6)	C8—C11—H11C	109.5
O2A—S1—C12	107.98 (13)	H11A—C11—H11C	109.5
C1—S1—C12	97.78 (11)	H11B—C11—H11C	109.5
C7—O1—C8	106.35 (18)	C13—C12—C17	111.8 (2)
C8—C1—C2	106.9 (2)	C13—C12—S1	112.15 (18)
C8—C1—S1	125.76 (19)	C17—C12—S1	107.30 (17)
C2—C1—S1	127.31 (18)	C13—C12—H12	108.5
C7—C2—C3	119.2 (2)	C17—C12—H12	108.5
C7—C2—C1	104.7 (2)	S1—C12—H12	108.5
C3—C2—C1	136.1 (2)	C12—C13—C14	110.8 (2)
C4—C3—C2	115.2 (2)	C12—C13—H13A	109.5
C4—C3—C9	122.7 (2)	C14—C13—H13A	109.5
C2—C3—C9	122.1 (2)	C12—C13—H13B	109.5
C3—C4—C5	125.2 (2)	C14—C13—H13B	109.5
C3—C4—Br1	118.04 (19)	H13A—C13—H13B	108.1
C5—C4—Br1	116.76 (18)	C15—C14—C13	111.2 (2)
C6—C5—C4	117.9 (2)	C15—C14—H14A	109.4
C6—C5—C10	120.1 (2)	C13—C14—H14A	109.4
C4—C5—C10	122.0 (2)	C15—C14—H14B	109.4
C7—C6—C5	117.9 (2)	C13—C14—H14B	109.4
C7—C6—H6	121.1	H14A—C14—H14B	108.0
C5—C6—H6	121.1	C14—C15—C16	110.9 (2)
O1—C7—C6	124.6 (2)	C14—C15—H15A	109.5
O1—C7—C2	110.9 (2)	C16—C15—H15A	109.5
C6—C7—C2	124.5 (2)	C14—C15—H15B	109.5
C1—C8—O1	111.1 (2)	C16—C15—H15B	109.5
C1—C8—C11	134.8 (2)	H15A—C15—H15B	108.0
O1—C8—C11	114.1 (2)	C15—C16—C17	111.6 (2)
C3—C9—H9A	109.5	C15—C16—H16A	109.3
C3—C9—H9B	109.5	C17—C16—H16A	109.3
H9A—C9—H9B	109.5	C15—C16—H16B	109.3
C3—C9—H9C	109.5	C17—C16—H16B	109.3
H9A—C9—H9C	109.5	H16A—C16—H16B	108.0
H9B—C9—H9C	109.5	C16—C17—C12	110.3 (2)
C5—C10—H10A	109.5	C16—C17—H17A	109.6
C5—C10—H10B	109.5	C12—C17—H17A	109.6
H10A—C10—H10B	109.5	C16—C17—H17B	109.6
C5—C10—H10C	109.5	C12—C17—H17B	109.6
H10A—C10—H10C	109.5	H17A—C17—H17B	108.1
			
O2B—S1—C1—C8	−120.2 (7)	C8—O1—C7—C2	−1.0 (3)
O2A—S1—C1—C8	−8.9 (3)	C5—C6—C7—O1	179.1 (2)
C12—S1—C1—C8	103.5 (2)	C5—C6—C7—C2	−0.8 (4)
O2B—S1—C1—C2	58.4 (7)	C3—C2—C7—O1	−177.3 (2)
O2A—S1—C1—C2	169.7 (2)	C1—C2—C7—O1	1.4 (3)
C12—S1—C1—C2	−77.8 (2)	C3—C2—C7—C6	2.7 (4)
C8—C1—C2—C7	−1.3 (3)	C1—C2—C7—C6	−178.6 (2)
S1—C1—C2—C7	179.92 (17)	C2—C1—C8—O1	0.7 (3)
C8—C1—C2—C3	177.1 (3)	S1—C1—C8—O1	179.52 (16)
S1—C1—C2—C3	−1.7 (4)	C2—C1—C8—C11	179.9 (3)
C7—C2—C3—C4	−2.7 (3)	S1—C1—C8—C11	−1.2 (4)
C1—C2—C3—C4	179.2 (2)	C7—O1—C8—C1	0.2 (3)
C7—C2—C3—C9	176.6 (2)	C7—O1—C8—C11	−179.2 (2)
C1—C2—C3—C9	−1.6 (4)	O2B—S1—C12—C13	160.6 (7)
C2—C3—C4—C5	1.1 (3)	O2A—S1—C12—C13	47.9 (2)
C9—C3—C4—C5	−178.1 (2)	C1—S1—C12—C13	−66.1 (2)
C2—C3—C4—Br1	−179.22 (16)	O2B—S1—C12—C17	37.5 (7)
C9—C3—C4—Br1	1.5 (3)	O2A—S1—C12—C17	−75.2 (2)
Br1^i^—Br1—C4—C3	−175.07 (14)	C1—S1—C12—C17	170.76 (17)
Br1^i^—Br1—C4—C5	4.6 (2)	C17—C12—C13—C14	−55.7 (3)
C3—C4—C5—C6	0.7 (4)	S1—C12—C13—C14	−176.3 (2)
Br1—C4—C5—C6	−179.00 (18)	C12—C13—C14—C15	55.8 (4)
C3—C4—C5—C10	179.6 (2)	C13—C14—C15—C16	−56.1 (4)
Br1—C4—C5—C10	0.0 (3)	C14—C15—C16—C17	56.3 (4)
C4—C5—C6—C7	−0.8 (3)	C15—C16—C17—C12	−55.5 (3)
C10—C5—C6—C7	−179.8 (2)	C13—C12—C17—C16	55.4 (3)
C8—O1—C7—C6	179.0 (2)	S1—C12—C17—C16	178.79 (19)

Symmetry code: (i) −*x*−1, −*y*+2, −*z*.

### Hydrogen-bond geometry (Å, º)

Cg1 is the centroid of the C2–C7 benzene ring.

**Table:**

*D*—H···*A*	*D*—H	H···*A*	*D*···*A*	*D*—H···*A*
C12—H12···O2*A*^ii^	1.00	2.44	3.355 (3)	151
C11—H11*C*···*Cg*1^iii^	0.98	2.83	3.547 (3)	130

Symmetry codes: (ii) *x*−1, *y*, *z*; (iii) *x*+1, *y*, *z*.
